# No-Touch Sequential Saphenous Venous Harvesting Technique in Off-Pump Bypass Surgery: A Retrospective Study

**DOI:** 10.3389/fcvm.2021.804739

**Published:** 2022-01-24

**Authors:** Xuejian Hou, Kui Zhang, Taoshuai Liu, Yang Li, Yang Zhao, Bangrong Song, Zhuhui Huang, Jubing Zheng, Ran Dong

**Affiliations:** Department of Cardiac Surgery, Beijing Anzhen Hospital, Capital Medical University, Beijing, China

**Keywords:** coronary artery bypass grafting (CABG), no touch technique (NT), off-pump CABG, sequential saphenous vein grafting, conventional saphenous vein graft harvesting

## Abstract

**Background:**

In the mid-1990s, the Swedish expert team proposed saphenous vein graft (SVG) harvesting with pedicle tissue. The short-term and long-term patency rates of the great saphenous vein obtained by the no-touch (NT) were higher than those obtained by the conventional (CON). In the past, NT harvesting was mainly used in on-pump coronary artery bypass grafting (CABG), and vein grafts were mostly single vein grafts. In this study, we retrospectively analyzed the safety and effectiveness of sequential vein grafts using NT harvesting in off-pump CABG.

**Methods:**

From 2017 to 2019, a total of 505 patients were included in the study. There were 150 patients in the NT group and 355 patients in the CON group. After applying propensity score matching (1:1 matching), 148 patients were included in each group. Baseline data, graft patency, post-operative complications, leg wound complications and 1-year major adverse cardiac and cerebrovascular events (MACCEs) were compared between the two groups.

**Results:**

There was no significant difference in the patency rate of sequential venous grafts between the two groups 1 year after the operation either before [NT: 7.1% (10/141) vs. CON: 11.5% (38/331), *p* = 0.149) or after matching (NT: 7.1% (10/140) vs. CON: 7.3% (9/124), *p* = 0.971]. There was no significant difference in the composite clinical endpoint between the two groups either before [NT: 3 (2.3%) vs. CON: 9 (2.8%), *p* = 1.000] or after matching [NT: 3 (2.3%) vs. CON: 3 (2.5%), *p* = 1.000]. There were differences in leg wound complications between the two groups both before [NT: 9 (6.9%) vs. CON: 6 (1.9%), *p* = 0.007] and after matching [NT: 9 (6.9%) vs. CON: 2 (1.7%), *p* = 0.043].

**Conclusions:**

The application of the NT harvesting in off-pump CABG with sequential vein grafts is safe and effective. NT method has disadvantages in leg wound.

## Introduction

Coronary artery disease is a serious threat to human health, especially complex coronary artery disease. At present, CABG is a good remedy for coronary artery diseases that are difficult to manage with interventional treatment. For CABG, the short-term and long-term patency rates are closely related to quality of life (1, 2). Additionally, the patency rates of grafts are closely related to the choice of vascular materials, of which the internal mammary artery is undoubtedly the best (3). An increasing number of studies have shown that the patency rate of the radial artery is also considerable (4). However, arterial materials have some disadvantages, such as easy spasm, limited length, and high occlusion rate when the target vessel stenosis is <90% (5). Therefore, the proportion of procedures using the great saphenous vein remains high. However, the great saphenous vein has relatively low short-term and long-term patency rates is prone to occlusion, so it is important to determine ways to improve the patency rate of vein grafts (6). In the mid-1990s, a team of Swedish experts proposed harvesting the great saphenous vein with pedicle tissue, that is, retaining part of the surrounding tissue in the process of harvesting the great saphenous vein and not expanding the vein manually after harvesting (7). The pedicled SV grafts are probably more durable than skeletonized (conventional) venous segments. They also conducted a short-term and long-term follow-up study that showed that the short-term and long-term patency rates of the great saphenous vein obtained by NT harvesting were higher than those obtained by the conventional procedure, especially in the long-term follow-up (8, 9). Some studies have showed that the NT grafts have excellent patency similar to that of radial artery (RA) grafts in long-term (10). NT technology is undoubtedly of great help in improving the patency rate of venous grafts (11–13).

As technological innovations continue to be developed, CABG has been increasingly performed in off-pump mode, which not only allows for faster patient recovery but also results in fewer post-operative complications (14). In addition, many studies have shown that there is no substantial difference between single vein and sequential vein grafts (15–17). Previous studies mainly used NT technology in on-pump CABG, and most of the vein grafts were single vein grafts. Therefore, this study retrospectively analyzed the safety and effectiveness of sequential vein grafts harvested by NT technology in off-pump CABG.

## Methods

### Patient Characteristics

**Figure 1:**
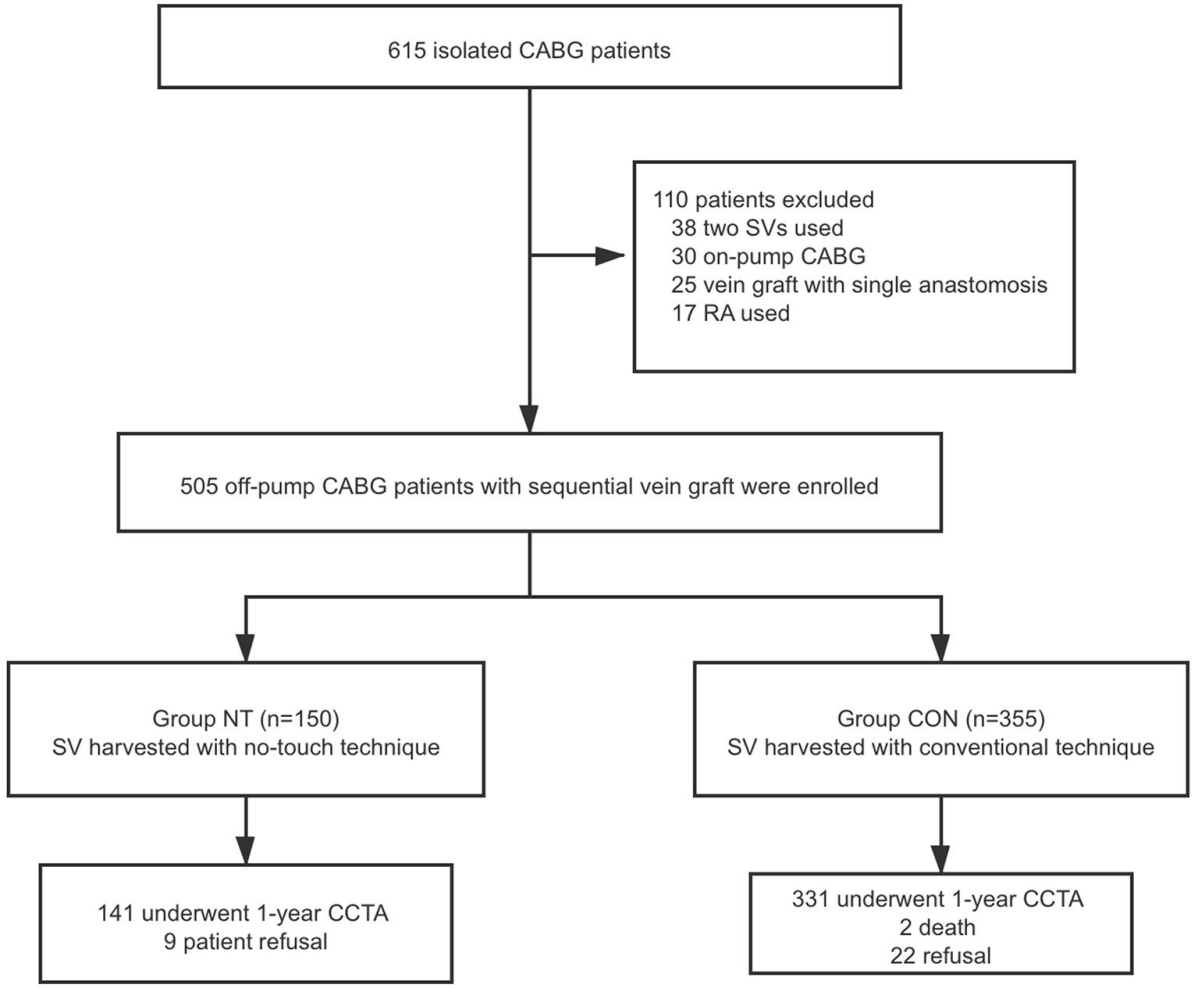
Summary flow diagram of enrolled patients. CABG, coronary artery bypass graft; SV, saphenous vein; RA, radial artery; CCTA, cardiac computed tomography angiography; NT, no touch technique; CON, conventional technique.

From 2017 to 2019, 615 patients were selected, including 165 patients treated with NT technology and 450 patients with conventional technology. Finally, a total of 505 patients were included in the study. As shown in Figure 1, a total of 150 patients were enrolled in the NT group, and 355 patients were enrolled in the CON group. The baseline data of the two groups were compared. There were differences in sex, body mass index (BMI), smoking, hypertension, previous percutaneous coronary intervention (PCI) history and New York Heart Association (NYHA) classification between the two groups. After applying propensity score matching (1:1 matching), 148 patients were included in each group. There was no significant difference in the baseline data of the matched groups, as shown in Table 1.

**Table 1:** Preoperative characteristics and risk factors of study patients.

**Variables**	**All study patients**			**Propensity-matched patients**		
	**Group NT (*n* = 150)**	**Group CON (*n* = 350)**	***P*-value**	**Group NT (*n* = 148)**	**Group CON (*n* = 148)**	***P*-value**
Age (years), mean ± SD	59.8 ± 9.0	60.5 ± 9.0	0.423	59.9 ± 8.9	60.4 ± 8.6	0.605
Female, *n* (%)	13 (8.7)	76 (21.4)	0.001	13 (8.8)	16 (10.8)	0.557
BMI > 25 (kg/m^2^), *n* (%)	94 (62.7)	181 (51.0)	0.016	92 (62.2)	90 (60.8)	0.811
Smoking, *n* (%)	73 (48.7)	137 (38.6)	0.036	72 (48.6)	83 (56.1)	0.20
Hypertension, *n* (%)	100 (66.7)	176 (49.6)	<0.001	98 (66.2)	100 (67.6)	0.805
Diabetes mellitus, *n* (%)	63 (42.0)	121 (34.1)	0.091	62 (41.9)	70 (47.3)	0.350
Previous MI, *n* (%)	57 (38.0)	128 (36.1)	0.679	57 (38.5)	60 (40.5)	0.721
Previous PCI, *n* (%)	23 (15.3)	32 (9.0)	0.037	22 (14.9)	19 (12.8)	0.614
Stroke, *n* (%)	16 (10.7)	30 (8.5)	0.429	16 (10.8)	16 (10.8)	1.000
NYHA, *n* (%)			<0.001			0.485
I	2 (1.5)	3 (0.8)		2 (1.4)	2 (1.4)	
II	109 (72.7)	161 (45.4)		107 (72.3)	101 (68.2)	
III	36 (24.0)	182 (51.3)		36 (24.3)	43 (29.1)	
IV	3 (2.0)	9 (2.5)		3 (2.0)	2 (1.4)	
LVEF (%) <45%, *n* (%)	6 (4.0)	20 (5.6)	0.448	6 (4.1)	11 (7.4)	0.212
Left main disease, *n* (%)	51 (34.0)	102 (28.7)	0.239	50 (33.8)	49 (33.1)	0.902

*NT, No-touch saphenous vein graft harvesting; CON, Conventional saphenous vein graft harvesting; BMI, Body mass index; MI, Myocardial infarction; PCI, Percutaneous coronary intervention; NYHA, New York Heart Association; LVEF, Left ventricular ejection fraction*.

### Operative Strategies

#### NT Group

**Figure 2:**
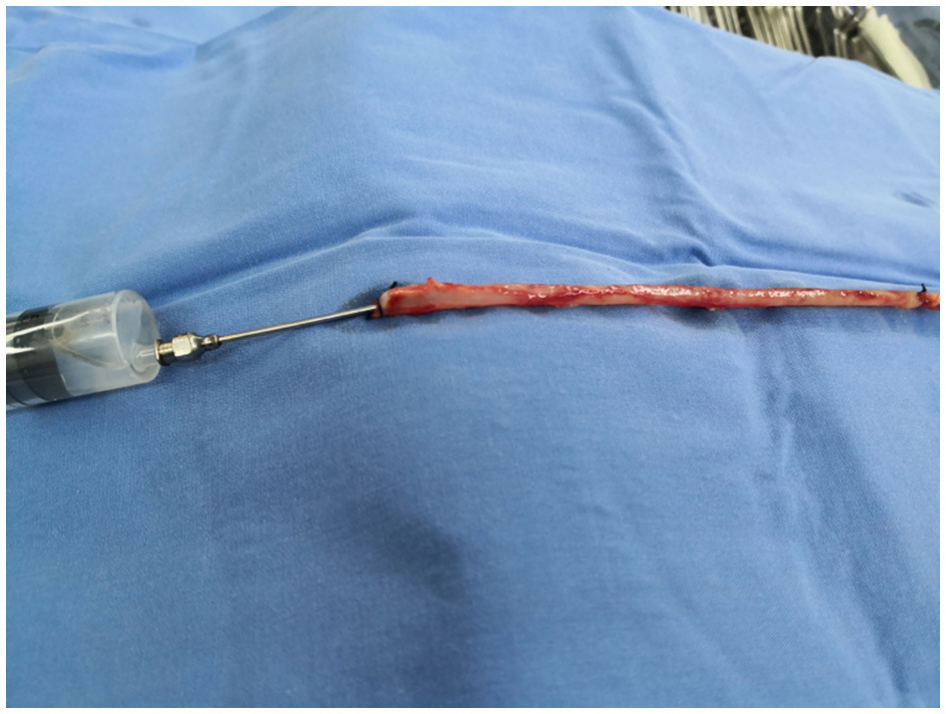
Conventional technology.

When harvesting the great saphenous vein, ~0.5 cm of tissue on both sides of the main vein was preserved without destroying the adventitia. The visible branches of the main vein were ligated with ligation wire, and the left and right sides were clamped with silver clips, as shown in Figure 2. After the vein was obtained, it was stored in a mixture of heparin and papaverine without manual dilation. After anastomosing with the proximal end of the ascending aorta, the blood pressure of the ascending aorta was used to check whether there was branch leakage in the main vein. If so, silver clips were used for clamping. After all anastomoses were completed, the sequential venous graft was checked again for blood leakage.

#### CON Group

**Figure 3:**
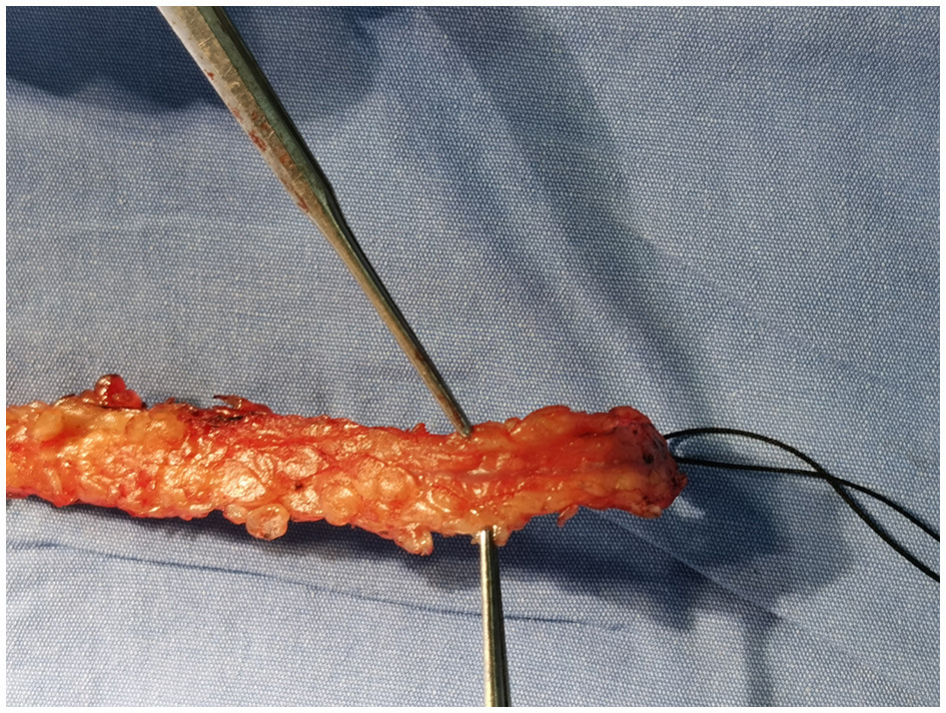
No-touch technology.

When obtaining the great saphenous vein, the surrounding tissue was not preserved, and the branches were treated in the same way as in the NT group. After harvesting, the vein was manually dilated with a syringe filled with heparin saline to check for branch leakage, as shown in Figure 3. The remaining operation procedures were the same as those used for the NT group.

### CCTA Evaluation of Graft Patency

One year after the operation, cardiac computed tomography angiography (CCTA) was performed to evaluate the patency rate of the sequential venous grafts. The venous patency rate was evaluated by the FitzGibbon classification system (18). FitzGibbon-A refers to a wide range of unobstructed grafts or <50% narrow grafts; FitzGibbon-B is a limited flow graft with a narrowing higher than 50%. FitzGibbon-O refers to an occlusive graft without blood flow. In this study, FitzGibbon-A/B was used for patency, and FitzGibbon-O was used for graft failure. The diseased graft was also regarded as a lesion if the lesion was located at the proximal/distal anastomosis site or the graft trunk.

### Evaluation of Clinical Outcomes

First, the incidence of post-operative complications, such as atrial fibrillation, acute kidney injury, and reoperation, was compared between the two groups. In addition, leg wound complications 3 months after the operation and the occurrence of MACCEs 1 year after the operation were evaluated.

### Statistical Analysis

SPSS 22.0 for Mac (IBM SPSS Statistics) was used for statistical analyses. Continuous variables are reported as the mean ± standard deviation or median (interquartile range) (IQR). Categorical variables were reported as the absolute frequency and as a percentage. Student's *t*-test was applied for continuous data with equal or unequal variances. The Mann-Whitney U test was applied for continuous data that were not normally distributed. Pearson's χ^2^ and Fisher's exact tests were used for categorical data. Statistical significance was accepted at *p* < 0.05.

## Results

### One Year CCTA Results

**Figure 4:**
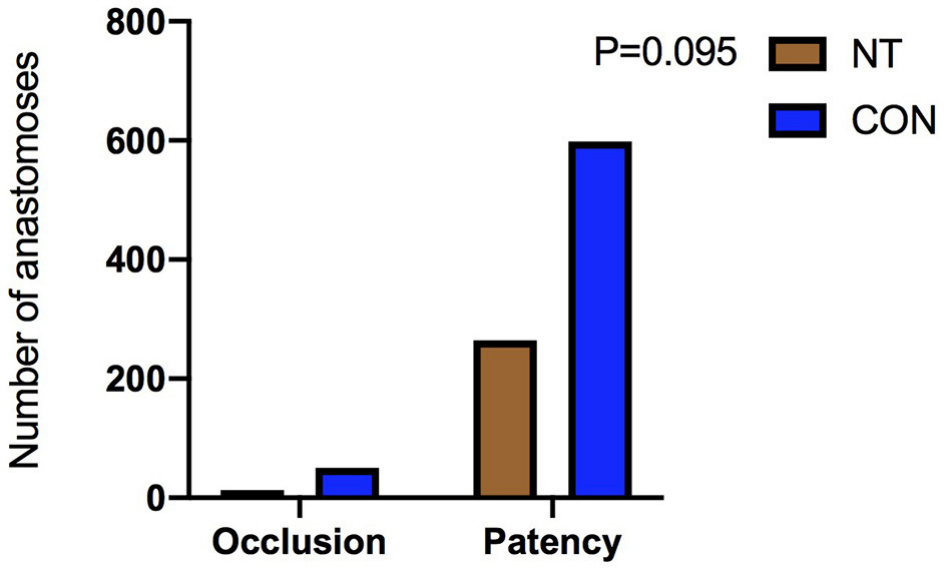
Comparison of patency rate before matching.

**Figure 5:**
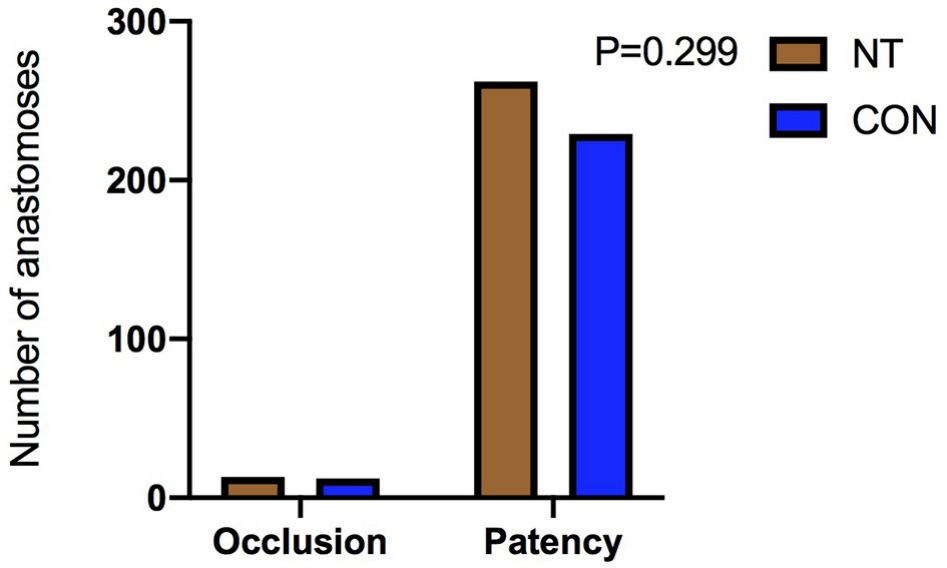
Comparison of patency rate after matching.

There was no significant difference in the patency rates of the sequential vein grafts, internal mammary artery grafts or total grafts between the two groups 1 year after the operation [before matching: sequential vein grafting, NT: 7.1% (10/141) vs. CON: 11.5% (38/331), *p* = 0.149; internal mammary artery grafting, NT: 1.5% (2/136) vs. CON: 3.8% (12/317), *p* = 0.313; total grafting, NT: 4.7% (13/277) vs. CON: 7.7% (50/648), *p* = 0.095, as shown in Figure 4; after matching: sequential vein grafting, NT: 7.1% (10/140) vs. CON: 7.3% (9/124), *p* = 0.971; internal mammary artery grafting, NT: 1.5% (2/135) vs. CON: 2.6% (3/117), *p* = 0.666; total grafting, NT: 7.4% (13/275) vs. CON: 5.0% (12/241), *p* = 0.299], as shown in Figure 5. In addition, the patency rates of the left anterior descending branch (LAD), left circumflex branch (LCX) and right coronary artery (RCA) territories were not significantly different between the two groups, as shown in Table 2.

**Table 2:** 1-year CCTA patency rates of the grafts and the coronary artery territories.

	**All study patients**			**Propensity-matched patients**		
**Grafts**	**Group NT (*n* = 150)**	**Group CON (*n* = 355)**	***P*-value**	**Group NT (*n* = 148)**	**Group CON (*n* = 148)**	***P*-value**
1-year patency	*n* = 141	*n* = 331		*n* = 140	*n* = 124	
Overall	4.7 (13/277)	7.7 (50/648)	0.095	7.4 (13/275)	5.0 (12/241)	0.299
Left ITA	1.5 (2/136)	3.8 (12/317)	0.313	1.5 (2/135)	2.6 (3/117)	0.666
Saphenous vein	7.1 (10/141)	11.5 (38/331)	0.149	7.1 (10/140)	7.3 (9/124)	0.971
LAD territory	1.7 (3/176)	3.5 (17/488)	0.309	1.1 (2/174)	1.2 (2/165)	1.000
LCX territory	1.5 (2/132)	2.6 (7/273)	0.755	1.5 (2/132)	1.9 (2/103)	1.000
RCA territory	6.0 (9/151)	10.5 (35/333)	0.107	6.0 (9/149)	7.6 (10/131)	0.597

*Values are % (n/N)*.

*CCTA, Cardiac computed tomography angiography; ITA, Internal thoracic artery; LAD, Left anterior descending branch; LCX, Left circumflex branch; RCA, Right coronary artery*.

### Early Clinical Hospital Outcomes

There was no difference in atrial fibrillation, acute kidney injury, reoperation or intra-aortic balloon pump (IABP) implantation between the two groups. Before matching, there were differences in the number of blood transfusions [NT: 15 (10.0%) vs. CON: 98 (27.6%), *p* < 0.001], ventilation time [NT: 17.0 (13.0, 21.6) vs. CON: 19.0 (15.0, 27.0), *p* < 0.001], and intensive care unit (ICU) stay [NT: 25.1 (20.0, 42.0) vs. CON: 34.1 (22.0, 50.2), *p* < 0.001]. After matching, there was no difference in ventilator time [NT: 17.0 (12.9, 21.4) vs. CON: 17.0 (14.0, 20.4), *p* = 0.398] or ICU stay [NT: 24.4 (20.0, 42.0) vs. CON: 23.0 (20.2, 39.7), *p* = 0.693]. However, differences remained in the number of blood transfusions between the two groups [NT: 15 (10.1%) vs. CON: 37 (25.0%), *p* = 0.001], as shown in Table 3.

**Table 3:** Comparison of early results in hospital.

**Variables**	**All study patients**			**Propensity-matched patients**		
	**Group NT (*n* = 150)**	**Group CON (355)**	***P*-value**	**Group NT (148)**	**Group CON (*n* = 148)**	***P*-value**
Atrial fibrillation, *n* (%)	44 (29.3)	99 (27.9)	0.742	43 (29.1)	49 (33.1)	0.451
Acute kidney injury, *n* (%)	10 (6.7)	40 (11.3)	0.114	10 (6.8)	15 (10.1)	0.296
Reoperation, *n* (%)	2 (1.3)	8 (2.3)	0.742	2 (1.4)	3 (2.0)	0.652
IABP implantation, *n* (%)	10 (6.7)	23 (6.5)	0.938	10 (6.8)	5 (3.4)	0.185
Blood transfusion, *n* (%)	15 (10.0)	98 (27.6)	<0.001	15 (10.1)	37 (25.0)	0.001
Ventilation time (h), median (IQR)	17.0 (13.0,21.6)	19 (15.0, 27.0)	<0.001	17.0 (12.9, 21.4)	17.0 (14.0, 20.4)	0.398
ICU stay (h), median (IQR)	25.1 (20.0, 42.0)	34.1 (22.0, 50.2)	<0.001	24.4 (20.0, 42.0)	23.0 (20.2, 39.7)	0.693

*IABP, Intra-aortic balloon pump; IQR, Interquartile range; ICU, Intensive care unit*.

### Main Clinical 1-Year Outcomes

There was no significant difference in the composite clinical end points between the two groups before matching [NT: 3 (2.3%) vs. CON: 9 (2.8%), *p* = 1.000] or after matching [NT: 3 (2.3%) vs. CON: 3 (2.5%), *p* = 1.000]. However, there were differences in leg wound complications 3 months after the operation between the two groups, both before [NT: 9 (6.9%) vs. CON: 6 (1.9%), *p* = 0.007] and after matching [NT: 9 (6.9%) vs. CON: 2 (1.7%), *p* = 0.043], as shown in Table 4. However, there was no significant difference between the two groups in leg wound complications after 1-year follow-up.

**Table 4:** Main outcomes and adverse event at 1-year.

**Variable**	**All study patients**			**Propensity-matched patients**		
	**Group NT (*n* = 150)**	**Group CON (*n* = 355)**	***P*-value**	**Group NT (*n* = 148)**	**Group CON (*n* = 148)**	***P*-value**
1-year outcomes	*n* = 130	*n* = 320		*n* = 130	*n* = 120	
Composite of MACCEs, *n* (%)	3 (2.3)	9 (2.8)	1.000	3 (2.3)	3 (2.5)	1.000
Death from any cause, *n* (%)	0 (0)	2 (0.6)	1.000	0 (0)	1 (0.8)	0.48
Myocardial infarction, *n* (%)	0 (0)	1 (0.3)	1.000	0 (0)	0 (0)	–
Stroke, *n* (%)	2 (1.5)	6 (1.9)	1.000	2 (1.5)	2 (1.7)	1.000
Repeat revascularization, *n* (%)	1 (0.8)	0 (0)	0.289	1 (0.8)	0 (0)	1.000
Leg wound complications, *n* (%)	9 (6.9)	6 (1.9)	0.007	9 (6.9)	2 (1.7)	0.043

*MACCEs, Major adverse cardiac and cerebrovascular events*.

## Discussion

This study retrospectively analyzed the application of the no-touch technique in off-pump bypass surgery with sequential vein grafts. The results indicate that this method is safe and effective. We compared the patency rate and MACCEs for sequential vein grafts harvested by the NT technique and conventional technique 1 year after the operation. The results showed that there was no significant difference between the two groups, suggesting that the use of sequential vein grafts harvested by the NT method in off-pump bypass is reasonable.

**Figure 6:**
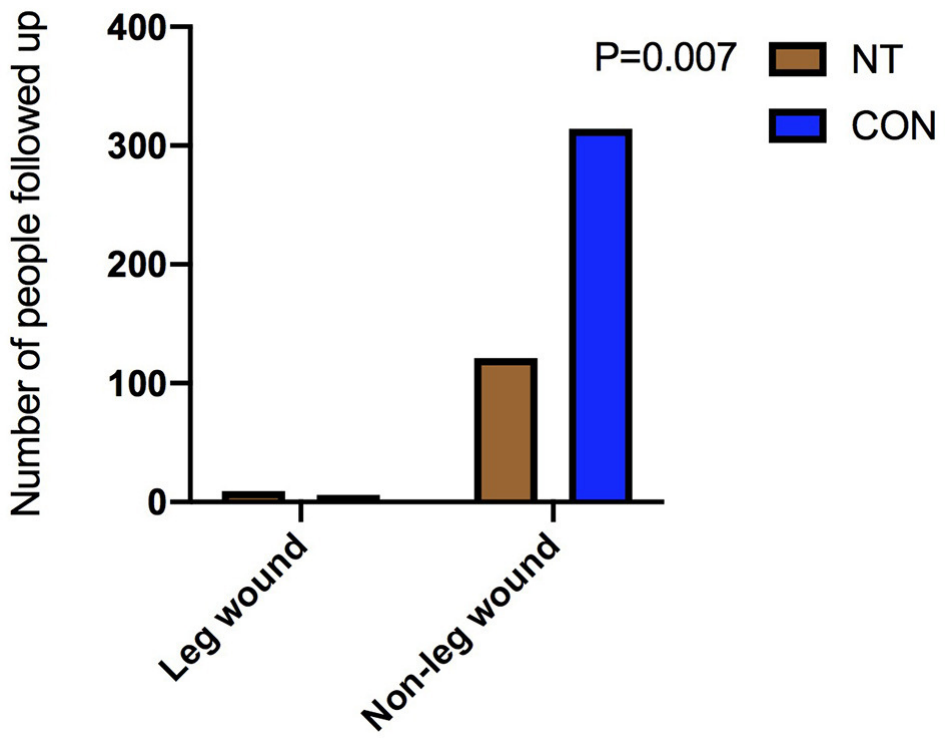
Comparison of leg wounds before matching.

**Figure 7:**
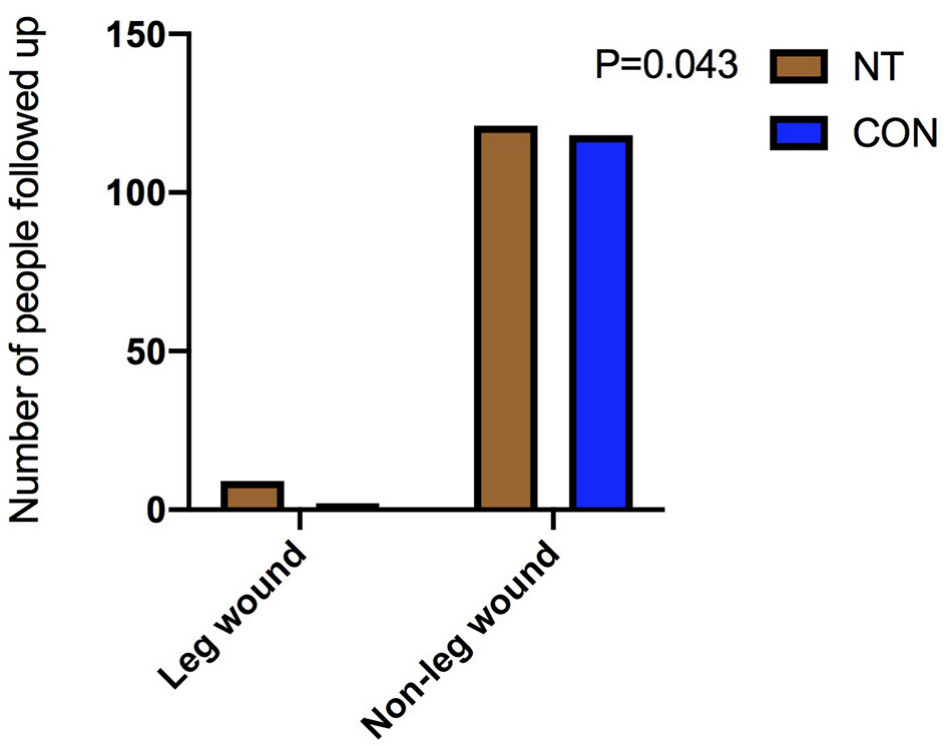
Comparison of leg wounds after matching.

Our results suggest that NT harvesting is superior to conventional method in terms of post-operative blood transfusion. The difference in post-operative blood transfusion between the two groups is not due to blood loss from venous tissue, but when the hemoglobin of post-operative patients is low (generally <7.0g/L). The difference in post-operative blood transfusion between the two groups is not entirely due to leg bleeding. However, regarding leg wound complications 3 months after surgery, NT harvesting was disadvantageous compared with conventional way as shown in Figures 6, 7. It is also easy to understand that some tissues, such as some small nutrient vessels and fat, are dissociated in the process of NT-based harvesting, which increases the duration of the leg wound healing process. Our findings are similar to those of previous studies. The incidence of poor wound healing after the NT harvesting is higher than that of the conventional great saphenous vein harvesting (9). In some previous studies (19), a drainage tube was used in the wound of the great saphenous vein after the vein was harvested by NT harvesting. Their results indicate that there was no significant difference in the occurrence of wound complications after drainage tube placement between NT and conventional method. This provides good guidance for the treatment of leg wounds after harvesting the great saphenous vein with NT method.

Previous studies showed that the vein grafts obtained by NT harvesting were mostly single vein grafts, and the most common surgery was on-pump CABG (7, 9, 20, 21). This is different from our study; we used all sequential vein grafts, and all procedures were off-pump CABG. Sequential vein grafts can preserve vein length, and off-pump bypass grafting can accelerate post-operative recovery and reduce the incidence of post-operative complications. In our study, there was no significant difference in the patency rate of sequential venous grafts between the two groups after 1 year of CCTA follow-up. In addition, there was no significant difference in the incidence of MACCEs between the two groups at the 1-year follow-up. This is basically consistent with previous studies. It has been proven that the application of sequential vein grafts harvested by NT technology in off-pump bypass surgery is safe and effective. Long-term differences between the two still need to be followed up in the future.

As we know, many patients with coronary heart disease have peripheral vascular disease at the same time. Therefore, their vascular tissue is very fragile, it is easy to damage the venous branches during acquisition, and the surrounding tissues can not be fully nourished due to the poor peripheral vascular blood supply conditions. For such patients, the less tissue damage when obtaining leg veins, the more favorable it is for the healing of leg wounds. NT method is very suitable for patients with severe peripheral vascular diseases.

Our study is a retrospective study, which implies a certain selection bias. In addition, our follow-up time was not sufficiently long. In the Souza study, we can see that the advantages of NT harvesting are gradually reflected in the longer follow-up time. In the future, we will continue to follow the patients for 3, 5, 10 years or even longer. In addition, the selection of a larger sample size and use of prospective research should be implemented, which we will strive to achieve in the future.

With the increasing number of off-pump CABG procedures, the expectations for the long-term graft patency rate are increasing. The application of sequential venous grafts harvested by NT in off-pump CABG is worthy of exploration and may provide better surgical treatment for patients with coronary artery disease.

## Conclusions

The application of the NT harvesting in off-pump CABG with sequential vein grafts is safe and effective. NT method has disadvantages in leg wound.

## Data Availability Statement

The raw data supporting the conclusions of this article will be made available by the authors, without undue reservation.

## Ethics Statement

The studies involving human participants were reviewed and approved by Institutional Ethics Committee of Beijing Anzhen Hospital. The patients/participants provided their written informed consent to participate in this study. Written informed consent was obtained from the individual (s) for the publication of any potentially identifiable images or data included in this article.

## Author Contributions

RD and JZ were responsible for the design, supervision of the study, and revision of the manuscript. XH drafted the manuscript. ZH and KZ designed a statistical plan. YL and TL participated in the revision of the manuscript and the coordination of the study. YZ and BS participated in data acquisition. All authors read and agreed to the final manuscript.

## Funding

This work was supported by the National Natural Science Foundation of China (Grant Nos. 81570373 and 81770412). The funding units did not participate in the design of the study and the implementation of related measures.

## Conflict of Interest

The authors declare that the research was conducted in the absence of any commercial or financial relationships that could be construed as a potential conflict of interest.

## Publisher's Note

All claims expressed in this article are solely those of the authors and do not necessarily represent those of their affiliated organizations, or those of the publisher, the editors and the reviewers. Any product that may be evaluated in this article, or claim that may be made by its manufacturer, is not guaranteed or endorsed by the publisher.

## Abbreviations

SVG: Saphenous vein graft
NT: No touch technique
CON: Conventional technique
CABG: Coronary artery bypass grafting
MACCEs: Major adverse cardiac and cerebrovascular events
RA: Radial artery
BMI: Body mass index
PCI: Percutaneous coronary intervention
NYHA: New York Heart Association
CCTA: cardiac computed tomography angiography
IQR: Interquartile range
LAD: Left anterior descending branch
LCX: Left circumflex branch
RCA: Right coronary artery
IABP: Intra-aortic balloon pump
ICU: Intensive care unit.
